# Editorial: Cellular senescence in diabetes: from markers to mechanisms and therapies

**DOI:** 10.3389/fendo.2023.1345529

**Published:** 2023-12-18

**Authors:** Cristina Aguayo-Mazzucato, Jeeyeon Cha, Peter J. Thompson

**Affiliations:** ^1^ Joslin Diabetes Center, Harvard Medical School, Boston, MA, United States; ^2^ Division of Diabetes, Endocrinology and Metabolism, Department of Medicine, Vanderbilt University Medical Center, Nashville, TN, United States; ^3^ Department of Physiology and Pathophysiology, Rady Faculty of Health Sciences, University of Manitoba, Winnipeg, MB, Canada

**Keywords:** beta cells, diabetes mellitus, senescence, SASP (senescence-associated secretory phenotype), therapies

Cellular senescence is a durable cell cycle arrest and response to stress that increases with age. It leads to the development of hallmark attributes such as persistent DNA damage response, metabolic adaptation, apoptosis resistance, and a secretory phenotype (the Senescence-Associated Secretory Phenotype, SASP). Recent work has shown that senescence is involved in a variety of endocrine and metabolic disorders such as obesity, fatty liver, cardiovascular disease, and diabetes, including Type 1 Diabetes, Type 2 Diabetes, and monogenic diabetes. However, due to the highly heterogeneous nature of senescence and context specificity (such as cell-type and developmental stage), there are major gaps in our understanding. Some of these gaps are: 1) lack of understanding about the mechanisms leading to activation of senescence and senescent cell accumulation during the progression of metabolic diseases, 2) specific markers that accurately distinguish senescent cells from their non-senescent counterparts still need to be determined, and 3) therapeutics that can safely and effectively counteract or mitigate senescent cell accumulation in diabetes and metabolic diseases are being studied.

This Research Topic aims to examine the current status of knowledge on β-cell senescence, some of the mechanisms by which it develops, its relevance in health and disease, and its potential as a therapeutic target. The present Research Topic assembles two original research papers that explore the role of β-cell senescence in the generational transmission of metabolic dysfunction and the interaction of senescence with altered nutrient sensing, another hallmark of aging. Additionally, two review papers and one perspective consolidate the latest knowledge on β-cell senescence, mechanisms, and markers, examining strategies for targeting this population and proposing an interesting perspective on the potential role for senescence during pancreatic islet development.

Varghese and Dhawan underscore how senescence does not only underlie aging and disease development but also plays a vital role in embryogenesis, tissue regeneration, and repair of the pancreas. During embryologic development, growth and patterning are partly dependent on p21^Cip1^-driven senescence and SASP promotes macrophage chemotaxis to clear senescent cells and hence remodel tissues. Although this process is well-documented in the development of certain organs, it has not yet been described in pancreatic islet development. However, it is known that high rates of β-cell replication occur during postnatal growth, which could increase risk of DNA damage. The latter, in turn, is a well-known initiator of SASP which can recruit macrophages allowing only “fit” β-cells to persist and grow. This interesting hypothesis warrants further experimentation, given its important implications for β-cell mass and its response to metabolic challenges in adulthood.

Iwasaki et al. explore the relationship between two known hallmarks of aging: alteration in nutrient sensing through IGF1R, and senescence. IGF1R is a marker of aged β-cells, and the authors used two models of downregulation of Igf1r signaling: Ames Dwarf mice, which have growth hormone deficiency and therefore decreased levels of IGF1, and an inducible β-cell specific IGF1R knock-down model. In both models, β-cell senescence markers were decreased. This was accompanied by improved glucose clearance, insulin secretion and maintenance of cellular identity, which persisted in the metabolic stress of high fat diet. These results suggest different levels of senescence regulation which could derive in identification of unique therapeutic targets.

In the review by Cha et al., we provided an updated list of markers supported by literature to evaluate β-cell senescence. Although we expect this list to evolve with time, it is important to note that there is not one universal senescence marker that defines all senescent cells across tissues, aging and disease models, underscoring the complexity of this cellular state. We therefore provided a toolkit to assess β-cell senescence and recommend that several independent markers that identify different features of senescence are tested in parallel to establish the presence of senescent β-cells. Importantly, we also highlighted that there is heterogeneity in β-cell senescence, and emerging evidence supports the existence of different subpopulations of senescent β-cells depending on the context and model. A robust identification of these cell populations and strategies to distinguish between them can then allow the development and evaluation of interventions directed to clearing pathologic senescent β-cells. Several transgenic mouse models are discussed as are the most relevant pharmacological approaches directed at killing senescent cells (senolytics) or modulating their SASP (senomorphics).

Rubin de Celis and Bonner-Weir point out that while there might be benefits of deleting senescent cells, senolytics have the potential to reduce the mass of β-cells, which is already compromised in type 1 and type 2 diabetes. Additionally, the beneficial effects of senomorphics might be due to some off-target effect, such as decreasing blood glucose levels or local inflammation. Therefore, the authors suggest a third approach to target β-cell senescence through modulators of senescence that can act at different levels such as evading cell cycle arrest (mTOR, Nrf2, transcription factor E2F1), microRNAs, splicing factors or dietary factors (probiotics and fatty acids). As explained, polyunsaturated fatty acids (PUFAs) are particularly interesting candidates for regulating senescence as they have shown the capacity to attenuate markers of immunosenescence, inflammaging, and cytokine imbalance and are effective in metabolically stressed pancreatic β-cells.

This Research Topic also highlights the potential role of sex differences in β-cell senescence. Escalona et al., evaluated the potential role of β-cell senescence as one of the mechanisms by which parental obesity increases the risk of offspring obesity, insulin resistance, diabetes, and reproductive disorders in a sex-dependent manner. Whereas they documented a clear increase in senescence (marked by increased *Cdkn2a* expression, encoding p16Ink4a, in β-cells) after 18 weeks of high fat diet, these changes did not persist in the male progeny, who still showed metabolic impairment. Cha et al., discuss current knowledge of sexual dimorphism and disease risk and speculate which aspects of sex might underlie differences in observed senescence rates and manifestations between males and females. The most dramatic example of sexual dimorphism in β-cell senescence is in human and mouse carriers of a pathogenic variant S64F in the transcription factor MAFA independent of sex hormones. Proposed mechanisms including sex-dependent responses to DNA damage and the role of genes encoded on sex chromosomes in repair mechanisms are described.

In conclusion, β-cell senescence plays an important role in the development of diabetes and is a therapeutic target with great potential to delay or halt progression of the metabolic diseases.

We commend the authors for their work and their contributions to this topical area in β-cell biology.

## Author contributions

CA-M: Writing – original draft, Writing – review & editing. JC: Writing – review & editing. PT: Writing – review & editing.

